# Red blood cell transfusion in animal models of acute brain injuries: a systematic review protocol

**DOI:** 10.1186/s13643-021-01703-8

**Published:** 2021-06-14

**Authors:** Mathieu Laflamme, Hourmazd Haghbayan, Manoj M. Lalu, Ryan Zarychanski, François Lauzier, Amélie Boutin, Malcolm R. Macleod, Dean A. Fergusson, Lynne Moore, Olivier Costerousse, Jacques Lacroix, Cheryl Wellington, Jamie Hutchison, Alexis F. Turgeon, on behalf of the Canadian Traumatic Brain Injury Research Consortium

**Affiliations:** 1 CHU de Québec – Université Laval Research Center, Population Health and Optimal Health Practices Research Unit (Trauma-Emergency-Critical Care Medicine), Université Laval, Québec, QC Canada; 2 Department of Surgery, Division of Neurosurgery, CHU de Québec – Université Laval, Québec, QC Canada; 3 Division of Cardiology, London Health Sciences Centre, Western University, London, ON Canada; 4 Clinical Epidemiology Program, Ottawa Hospital Research Institute, Ottawa, ON Canada; 5 Regenerative Medicine Program, Ottawa Hospital Research Institute, Ottawa, ON Canada; 6 Department of Anesthesiology and Pain Medicine, The Ottawa Hospital, Ottawa, ON Canada; 7 Department of Internal Medicine, Sections of Critical Care Medicine, of Hematology and of Medical Oncology, Rady Faculty of Medicine, University of Manitoba, Winnipeg, MB Canada; 8 Research Institute of Oncology and Hematology, CancerCare Manitoba, Winnipeg, MB Canada; 9 Department of Anesthesiology and Critical Care Medicine, Division of Critical Care Medicine, Université Laval, Québec City, QC Canada; 10 Department of Medicine, Université Laval, Québec City, QC Canada; 11 Department of Pediatrics, Université Laval, Québec, QC Canada; 12 Centre for Clinical Brain Sciences, University of Edinburgh, Edinburgh, UK; 13 Department of Social and Preventive Medicine, Université Laval, Québec City, QC Canada; 14 Division of Pediatric Critical Care Medicine, Department of Pediatrics, Faculty of Medicine, Université de Montréal, Montréal, QC Canada; 15 Department of Pathology and Laboratory Medicine, University of British Columbia, Vancouver, BC Canada; 16 Djavad Mowafaghian Centre for Brain Health, University of British Columbia, Vancouver, BC Canada; 17 Department of Pediatrics, University of Toronto, Toronto, ON Canada

**Keywords:** Red blood cell transfusion, Blood products, Traumatic brain injury, Stroke, Cerebral hemorrhage, Animal models

## Abstract

**Background:**

Anemia is common in neurocritically ill patients. Considering the limited clinical evidence in this population, preclinical data may provide some understanding of the potential impact of anemia and of red blood cell transfusion in these patients. We aim to estimate the association between different transfusion strategies and neurobehavioral outcome in animal models.

**Methods:**

We will conduct a systematic review of comparative studies of red blood cell transfusion strategies using animal models of traumatic brain injury, ischemic stroke or cerebral hemorrhage. We will search MEDLINE, EMBASE, and Web of Science databases for eligible studies from inception onwards. Two independent reviewers will perform study selection and data extraction. We will report our results in a descriptive synthesis focusing on characteristics of included studies, reported outcomes, risk of bias, and construct validity. Our primary outcome is the neurological function (neurobehavioral performance) and our secondary outcomes include mortality, infarct size, intracranial pressure, cerebral perfusion pressure, cerebral blood flow, and brain tissue oxygen tension. If appropriate, we will also perform a quantitative synthesis and pool results using random-effect models. Heterogeneity will be expressed with *I*^2^ statistics. Subgroup analyses are planned according to animal model characteristics, co-interventions, and risks of bias.

**Discussion:**

Our study is aligned with the efforts to better understand the level of evidence on the impact of red blood cell transfusion strategies from preclinical studies in animal models of acute brain injury and the potential translation of information from the preclinical to the clinical research field.

**Systematic review registration:**

PROSPERO CRD42018086662.

**Supplementary Information:**

The online version contains supplementary material available at 10.1186/s13643-021-01703-8.

## Background

Anemia is frequently encountered in critically ill patients [1, 2] and red blood cell (RBC) transfusions are often used to correct anemia [1–3]. Over recent decades, data on the potential impact of RBC transfusion on mortality and other clinically significant outcomes have been collected from large cohort studies [1, 4, 5]. Large randomized controlled trials (RCTs) conducted across all critically ill populations support the use of restrictive RBC transfusion strategy [6, 7]. However, in brain injury, concerns have been raised that criteria for transfusion which require more severe anemia may increase the risk of brain hypoxia [8, 9].

Anemia is associated with a potential decrease in oxygen (O_2_) tissue delivery [10]. Under normal conditions, increased cerebral blood flow occurs to compensate for reduced arterial oxygen content (C_a_O_2_) [11, 12]. However, increased metabolic demand and a loss of compensation mechanisms in the injured brain may increase vulnerability to secondary injury caused by anemia [13–15]. In the last two decades, multiple observational studies have shown contradictory results when assessing the relation between anemia, RBC transfusion, and clinical outcomes in the neurocritically ill population [16–20]. The few RCTs on RBC transfusion in this specific population were also unable to show superiority of any transfusion strategy [9, 21, 22], confirming our inability to formulate recommendations on a specific transfusion strategy for patients with acute brain injury [23].

It is unclear whether previous clinical trials were designed on the basis of a robust assessment of findings from animal studies and the extent to which those studies may be relevant to human disease. Given the potential importance of data from preclinical trials in the elaboration of clinical studies and the importance of systematically reviewing the literature to ensure the best use of preclinical data for improving both experimental and clinical research [24], we will conduct a systematic review of comparative preclinical studies evaluating the effect of RBC transfusion strategies on preclinical outcomes in animal models of brain injury.

## Methods

### Design

This protocol is based on *The Cochrane Handbook for Systematic Reviews of Interventions* and specific recommendations for conducting systematic reviews and meta-analysis of preclinical studies [25, 26]. We will report our results according to the Preferred Reporting Items for Systematic Reviews and Meta-Analysis (PRISMA) [27]. The present protocol has been registered within the PROSPERO database (registration number CRD42018086662) and is being reported in accordance with the reporting guidance provided in the Preferred Reporting Items for Systematic Reviews and Meta-Analyses Protocols (PRISMA-P) statement [28, 29] (see checklist in Additional file 1).

### Eligibility criteria

We will include studies using in vivo animal models with acute cerebral lesions limited to traumatic brain injury (all types) or stroke (ischemic or hemorrhagic) comparing outcome in injured animals treated with a specific RBC transfusion strategy (restrictive or liberal) with outcome in injured animals treated with a different RBC transfusion strategy or no treatment or any other intervention. In our study, the hemorrhagic stroke group refers to animal models of intracerebral hemorrhage (ICH) or subarachnoid hemorrhage (SAH). Both animal models of focal and global brain ischemia will be included. There will be no restrictions in terms of species and comorbidities of models. We will consider studies regardless of their primary outcomes of interest. There will be no restriction applied to date or language of publication. We will consult translators for publications not written in English or French. Our structured research question and our study eligibility criteria are presented in Tables 1 and 2, respectively.

**Table 1:** Structured question

Population	Animal models of:
	- Traumatic brain injury
	- Ischemic stroke
	- Hemorrhagic stroke
	(ICH, SAH)
Intervention	Red blood cell transfusion
Comparator	An alternative red blood cell transfusion strategy (hemoglobin threshold/target) or No transfusion or Any other intervention
Primary outcomes	Neurobehavioral
Secondary outcomes	All-cause mortality Infarct size Intracranial pressure Cerebral perfusion pressure (CPP) Cerebral blood flow (CBF) Brain tissue oxygen tension (PbtO_2_)
Study design	Comparative preclinical studies

*ICH* intracerebral hemorrhage, *SAH* subarachnoid hemorrhage

**Table 2:** Eligibility criteria

Inclusion criteria	Exclusion criteria
1. Studies comparing at least two interventional groups	1. Human studies
2. In vivo animal models of TBI or ischemic stroke or hemorrhagic stroke (ICH or SAH)	2. In vitro studies
3. RBC transfusion in at least one interventional group	3. Non-mammals used as animal models
4. Different transfusion strategy or no transfusion or any other intervention as a comparator	4. Transfusion before cerebral injury is induced
5. Any study outcome	

*TBI* traumatic brain injury, *ICH* intracerebral hemorrhage, *SAH* subarachnoid hemorrhage, *RBC* red blood cell

### Study identification

We will search MEDLINE (PubMed), EMBASE, and Web of Science, from inception onwards, using a structured search strategy. We have developed a strategy for MEDLINE using a combination of keywords related to “anemia,” “red blood cell transfusion,” “traumatic brain injury,” “stroke,” and “intracranial hemorrhage.” We have also included the Medical Subject Headings (MeSH) terms linked to the keywords mentioned previously and a search filter for animal studies [30]. This search strategy has been pre-tested to obtain a high sensitivity and acceptable specificity. It was also reviewed by an information specialist with expertise in health sciences for additional robustness. The strategy was then modified for EMBASE (with Emtree and a specific filter for animal studies) and Web of Science [31]. The search strategy used for MEDLINE is presented in Additional file 2. We will also review references of the included studies identified through database searches to identify additional studies. We will search databases from their inception, and we will update the search prior to submission of the systematic review in order to include the most recent eligible studies.

### Study selection

Records resulting from electronic database searches will be imported in Endnote (version X8, New York City: Thomson Reuters, 2016) where duplicates will be identified and removed. Titles and abstracts of retrieved records will then be transferred to a Microsoft Excel (version 15.29, Redmond, WA: Microsoft, 2016) spreadsheet and screened independently by two reviewers. Full-text articles of potentially eligible studies will be retrieved and reviewed to assess full eligibility. Disagreement will be resolved by consensus. If consensus is not possible, a senior team member will arbitrate.

### Data collection

Data from included studies will be abstracted independently by two reviewers using a standardized and pretested form. In case of discrepancy, consensus will be reached through discussion or the involvement of a third reviewer. We will collect data on study characteristics (design, length of follow-up), sample characteristics (species, number, age, weight, gender, hemoglobin level), model preparation (anesthesia, type and severity of injury, hemorrhage, hemodilution), intervention (target, threshold, units, volume, timing with injury), co-interventions (crystalloids, colloids, medications, surgical interventions, etc.) and outcomes. We will use graph analysis tools to extract data of interest if results are not reported in the text or tables. If necessary, we will contact authors for additional information.

### Outcomes

Our primary outcome is neurological function (neurobehavioral performance). Some examples of neurobehavioral tests that we expect to encounter include, but are not limited to, cylinder test, beam balance, beam walking, rotarod test, open field test, Morris water maze, elevated T-maze, and passive avoidance. Our secondary outcomes include mortality, infarct size, intracranial pressure, cerebral perfusion pressure, cerebral blood flow, and brain tissue oxygen tension. We will consider all neurobehavioral tests reported in the included studies. We will collect information on the definition of mortality used in included studies, whether animals had died spontaneously or if they received euthanasia when they reached a state of imminent death according to specific criteria of deterioration of their condition.

### Risk of bias

We will use an adaptation of the Collaborative Approach to Meta Analysis and Review of Animal Data from Experimental Studies (CAMARADES) tool (See Additional file 3) for risk of bias assessment [32]. The same two reviewers who abstracted data will independently assess the risk of bias. The domains evaluated are (i) selection bias (inclusion and exclusion criteria, randomization), (ii) information bias (blinding of outcome assessment), (iii) confounding (blinding of induction to cerebral lesions and care during the follow-up, comorbidities, temperature control, anesthesia agents), and (iv) other bias or considerations (selective reporting, conflict of interest, sample size, peer-reviewed publications, animal welfare). The assessment of criteria for animals to enter the study and the assessment of selective reporting are the two elements that we added to the original tool.

### Quality of reporting

We will evaluate the quality of reporting in individual publications with a list of questions according to the NIH Principles and Guidelines for Reporting Preclinical Research [33]. These principles were elaborated with editors from more than 30 preclinical and basic science journals. Many journals have agreed to endorse these guidelines.

### Construct and external validity

Two reviewers will independently assess each included study for the potential applicability of the results to the clinical setting defined as construct validity. We will evaluate baseline characteristics of the animals (species, age, sex, comorbidities) as well as methods for model preparation (TBI, acute ischemic stroke, ICH, SAH, anemia), type of blood transfusion, definition of death and co-interventions. Setting of the studies (single vs multicentered) and heterogeneity of the animal population (age, sex, comorbidities, breeding) will help evaluate external validity (generalizability of the results) (Table 3).

**Table 3:** Construct and external validity

Domains	Description
Age	All same age vs different ages
Sex	All male vs all female vs mixed
Comorbidities	All healthy vs models with comorbidities vs mixed
Breeding	Inbred only vs outbred (wild-type) only vs mixed
Number of participating centers	Single vs multicentered
Species	Rats, mice, monkeys, cats, dogs, and others
TBI models	Fluid percussion injury (FPI), controlled cortical impact (CCI), weight-drop model, acceleration model
Acute stroke models	Spontaneous vs induced, global vs focal ischemia, endovascular vs open surgery with ligature, embolic, and photothrombosis
ICH models	Blood vs collagenase injection in cerebral parenchyma
SAH models	Endovascular perforation of internal carotid artery, blood injection in basal cisterns
Blood transfusion type	Whole blood vs packed red blood cells
Anemia	Hemorrhage or hemodilution as part of model preparation
Co-interventions	Crystalloids, colloids, medications, mechanical ventilation, and others
Timing of treatment	Immediate vs delayed treatment measures after brain injury
Death	Animals found dead or sacrificed when met criteria for important deterioration (imminent death)

### Data analysis and synthesis

A descriptive synthesis of our results will be presented. We plan to report data on neurobehavioral performance, and any other continuous data, with normalized mean difference (NMD) as the effect-size measure with 95% confidence intervals (CIs). This is considering that data from sham and uninjured animal models are available or can be easily inferred. If there are no data available on sham animals or it cannot be inferred, we will report neurobehavioral results with standardized mean difference (SMD). In a single study, different tests can be conducted to assess neurobehavioral performance in the same cohort of animals. To synthesize the overall neurobehavioral performance, we will combine data from neurobehavioral tests into a single “nested” outcome, when possible, using a method previously described by members of our research team (Vesterinen 2014) [34]. Mortality data, as well as any other dichotomous data, will be reported with risk ratios (RR) as the effect-size measure and 95% confidence intervals (95% CIs).

If appropriate, we will pool data from our primary and secondary outcomes using the inverse of the variance with random-effects models. We will need at least 3 studies using similar animal models, transfusion strategy and comparator to conduct a meta-analysis. Cochrane Review Manager (RevMan) version 5.3 (The Cochrane Collaboration, Copenhagen, Denmark, 2014) will be used. Statistical heterogeneity will be evaluated using the *I*^2^ index and classified as very low (0–25%), low (25–50%), moderate (50–75%), or high (> 75%) [35]. Funnel plots will be used for the evaluation of potential publication bias for neurobehavioral outcome and mortality results with additional analysis for each type of brain injury. Subgroup analyses will be conducted when possible using the following: mammal class orders (primates vs. rodents vs. all others), transfusion thresholds (restrictive vs. liberal strategies), the presence of co-interventions, induced anemia during preparation (hemorrhage vs. hemodilution vs none), type of neurocritical condition (TBI vs. ischemic stroke vs. ICH vs. SAH) and risk of bias (high/unclear risk of bias vs. low risk of bias).

## Discussion

The results of this systematic review will provide a robust, timely summary of the preclinical evidence relating to the effects of different transfusion practices in models of neurocritical illnesses. It will inform the design of future preclinical studies seeking to identify the optimal transfusion practice following acute brain injury including traumatic brain injury, ischemic stroke, and cerebral hemorrhage. The best use and review of evidence of existing preclinical data to inform clinical research is increasingly recommended from a study design perspective [24] but also for ethical, economic, and scientific principles [26, 36, 37]. Evaluating the risks of bias in preclinical studies will also indicate whether strategies for research improvement are needed in this field.

Over the years, many epidemiologists have been highly critical of preclinical data because of the lack of conformity with important methodological concepts [38]. A decade ago, results from previous systematic reviews of clinical data were compared with results of systematic reviews of preclinical data conducted on three interventions in neurocritical conditions (corticosteroids in brain injury, thrombolysis, and tirilazad in ischemic stroke) [39]. The observed results were discordant, a finding likely secondary to important bias in the preclinical animal studies and the inability of animal models and of the design of these preclinical studies to adequately reflect the clinical conditions. The two main methodological flaws identified were related to the randomization and the blinding of outcome assessments, which were rarely described [38]. Consequently, we do expect that a significant proportion of the included studies in our systematic review will present major concerns in terms of risk of bias related to lack of randomization and blinding.

Since our review will focus on neurobehavioral outcome and mortality in animal models, ethical concerns about animal welfare will need special consideration in the interpretation of results. Committees supervising animal research and responsible for giving ethical approval may require investigators to establish humane endpoints as criteria for euthanasia [40]. The use of death as an endpoint, however, may be justified when scientific validity cannot be achieved if the animals are sacrificed at any point in time before death or imminent death occurs. Fields of research using animal models of severe conditions such as toxicology and sepsis require death or near-death as an endpoint because even small differences in mortality can be a significant step towards more experimentation using a specific therapy [41]. However, it is important to note that animal ethics boards are increasingly discouraging studies using death as an endpoint. Thus, scientific validity cannot be achieved if the animals are sacrificed at any point in time before death or imminent death occurs, and we expect to find several studies using translational outcomes. This reality of preclinical research was the main argument for choosing neurobehavioral performance instead of mortality as our primary outcome. However, we will take care to extract and report if animals were sacrificed or found dead in included studies reporting data on mortality in animal models.

We expect heterogeneity in the methodology of eligible studies especially regarding the induction of brain injury, blood transfusion strategies, comparators, and outcome assessment. We also expect methodological limitations concerning the randomization process and the blinding of outcome assessment. To address these concerns, we may not be able to conduct overall pooled analyses but rather conduct quantitative analyses for studies that can be compared in terms of brain injury models, transfusion, and comparators. The potential impact of the risk of bias of studies will be evaluated through subgroup analyses. Finally, a systematic review of preclinical studies is a greater risk of overstating efficacy considering the high likelihood of publication bias [42].

We plan to disseminate our results at conference presentations and publication in a peer-reviewed journal. Any amendments made to this protocol when conducting the review will be outlined in PROSPERO and reported in the final manuscript.

In conclusion, we propose to conduct a systematic review that will synthesize the preclinical evidence of the impact of the use of RBC transfusion in animal models of acute brain injury. This systematic review will facilitate the translation of laboratory research to clinical trials.

## Supplementary Information

**Additional file 1.** PRISMA-P checklist.

**Additional file 2.** Search strategy for MEDLINE/PubMed. Description: This file contains the comprehensive search strategy we developed for the MEDLINE/PubMed database.

**Additional file 3.** Risk of bias evaluation form. Description: This file contains our tool for risk of bias assessment of preclinical studies adapted from CAMARADES.

## Data Availability

Not applicable.

## Abbreviations

RBC: Red blood cell
RCT: Randomized controlled trial
O_2_: Oxygen
CaO_2_: Arterial oxygen content
ICH: Intracerebral hemorrhage
SAH: Subarachnoid hemorrhage
TBI: Traumatic brain injury
RR: Risk ratio
CI: Confidence interval
NMD: Normalized mean difference
SMD: Standardized mean difference
