# Supplementary material for: Red blood cell transfusion in animal models of acute brain injuries: a systematic review protocol
Source: Syst Rev. 2021 Jun 14;10:177. doi: 10.1186/s13643-021-01703-8 (PMC8201673; doi:10.1186/s13643-021-01703-8)
Supplement: Supplementary file 3 — Additional file 3. Risk of bias evaluation form. Description: This file contains our tool for risk of bias assessment of preclinical studies adapted from CAMARADES. [file 13643_2021_1703_MOESM3_ESM.docx]

**Additional file 3: Risk of bias evaluation form**

| **Domain** | **Description** | **Judgment** | **Risk of bias** | **Direction** |
| --- | --- | --- | --- | --- |
| **Selection bias** | | | | |
| Inclusion and exclusion criteria | | | | |
| Were inclusion and exclusion criteria specified? | *.* | □ yes  □ no  □ unclear |  | □ overestimation  □ underestimation  □ unclear |
| Were inadequate eligibility criteria avoided? |  | □ yes  □ no  □ unclear |  | □ overestimation  □ underestimation  □ unclear |
| Randomization | | | | |
| Was randomization reported? |  | □ yes  □ no  □ unclear |  | □ overestimation  □ underestimation  □ unclear |
| Was the method specified and done after lesion? |  | □ yes  □ no  □ unclear |  | □ overestimation  □ underestimation  □ unclear |
| Overall |  |  | □ high  □ low  □ unclear | □ major overestimation  □ overestimation  □ underestimation  □ major underestimation  □ unclear |
| **Information bias** | | | | |
| Blinding of outcome assessment | | | | |
| Were outcome assessment blinded to treatment allocation? |  | □ yes  □ no  □ unclear |  | □ overestimation  □ underestimation  □ unclear |
| Overall |  |  | □ high  □ low  □ unclear | □ major overestimation  □ overestimation  □ underestimation  □ major underestimation  □ unclear |
| **Confounding** | | | | |
| Blinding of care | | | | |
| Were induction of cerebral lesions, maintenance, decision regarding care blinded to treatment allocation? |  | □ yes  □ no  □ unclear |  | □ overestimation  □ underestimation  □ unclear |
| Comorbidities | | | | |
| Were animals with comorbidities included? |  | □ yes  □ no  □ unclear |  | □ overestimation  □ underestimation  □ unclear |
| Temperature | | | | |
| Animal temperature controlled? |  | □ yes  □ no  □ unclear |  | □ overestimation  □ underestimation  □ unclear |
| Was the method to control temperature adequate? |  | □ yes  □ no  □ unclear |  | □ overestimation  □ underestimation  □ unclear |
| Anesthesia |  |  |  |  |
| Agents known for important neuroprotective activity? |  | □ yes  □ no  □ unclear |  | □ overestimation  □ underestimation  □ unclear |
| Overall |  |  | □ high  □ low  □ unclear | □ major overestimation  □ overestimation  □ underestimation  □ major underestimation  □ unclear |
| **Other treats to internal validity** | | | | |
| Conflict of interest | | | | |
| Was a statement of conflict of interest was reported? |  | □ yes  □ no  □ unclear |  | □ overestimation  □ underestimation  □ unclear |
| Did authors report a conflict of interest? |  | □ yes  □ no  □ unclear |  | □ overestimation  □ underestimation  □ unclear |
| Random error / Sampling error | | | | |
| Was the sample size calculation reported? |  | □ yes  □ no  □ unclear |  | □ overestimation  □ underestimation  □ unclear |
| Was the precision of the estimate sufficient? |  | □ yes  □ no  □ unclear |  | □ overestimation  □ underestimation  □ unclear |
| Internal publication bias | | | | |
| Are reports of the study free of suggestions of selective outcome reporting? |  | □ yes  □ no  □ unclear | □ high  □ low  □ unclear | □ overestimation  □ underestimation  □ unclear |
| Were outcomes and statistical analysis described in a protocol published a priori? |  | □ yes  □ no  □ unclear | □ high  □ low  □ unclear | □ overestimation  □ underestimation  □ unclear |
| **Other methodological quality concerns** | | | | |
| Peer review | | | | |
| Was the study report peer reviewed? |  | □ yes  □ no  □ unclear | □ high  □ low  □ unclear | □ overestimation  □ underestimation  □ unclear |
| Animal welfare | | | | |
| Was there a statement of compliance with animal welfare explicitly stated? |  | □ yes  □ no  □ unclear | □ high  □ low  □ unclear | □ overestimation  □ underestimation  □ unclear |
